# Superspace crystallography: a key to the chemistry and properties

**DOI:** 10.1107/S2052252514023550

**Published:** 2015-01-01

**Authors:** Carlos Basílio Pinheiro, Artem M. Abakumov

**Affiliations:** a Laboratório de Cristalografia, Departamento de Física, Universidade Federal de Minas Gerais - UFMG, Belo Horizonte, Brazil; b EMAT, University of Antwerp, Groenenborgerlaan 171, B-2020, Belgium; c Chemistry Department, Moscow State University, Moscow 119991, Russian Federation

**Keywords:** superspace crystallography, modulated structure, incommensurate modulation, chemistry

## Abstract

An overview is given of the recent advances in the field of modulated molecular and inorganic crystals with an emphasis on the links between incommensurability, intermolecular and interatomic interactions and, wherever possible, the properties of the materials. The importance of detailed knowledge on the modulated structure for understanding the crystal chemistry and the functional properties of modulated phases is shown using selected examples of incommensurate modulations in organic molecular compounds and inorganic complex oxides.

## Abstract 2

An overview is given to recent advances in the field of modulated molecular and inorganic crystals. The importance of detailed knowledge of the modulated structure to understand crystal chemistry and the functional properties of the modulated phases is outlined.

## 1. Introduction   

This review is focused on recent advances in the field of aperiodic molecular crystals and complex oxides. Taking into account the many reviews and books published on aperiodic materials, it is unnecessary to provide an extensive and comprehensive overview of the large number of modulated structures known so far in this relatively short paper. Instead, we address our review to a reader who is a chemist or a materials scientist rather than a crystallographer; thus placing the main accent on the relationship between incommensurate order, chemical bonding and, wherever possible, functional properties. This is in line with our feeling that in the context of the steadily growing variety of aperiodic crystal structures such relationships deserve particular attention. Nowadays, the emphasis is greatly shifted towards practical applications and, following this trend, we are willing to demonstrate that incommensurability is intrinsic in many popular materials and, moreover, their properties cannot be adequately interpreted without knowing details about their modulated structures and the peculiarities of interatomic interactions behind the incommensurate order. The review does not provide the basics of superspace crystallography, assuming that the reader can acquire them from other sources, such as the works by Janssen *et al.* (2006 ▶, 2007 ▶), van Smaalen (2004 ▶, 2007 ▶), Wagner & Schönleber (2009 ▶) and Janssen & Janner (2014 ▶). Nevertheless, we decided to include a brief overview of the historical background of the field, in order to let the reader feel the flavour of aperiodic crystallography. Further insight into the history of aperiodic crystallography can be found in articles by Janssen (2012 ▶) and Janssen & Janner (2014 ▶).

## 2. Historical background   

The notion that three-dimensional translational symmetry, *i.e.* periodicity, was an intrinsic property of crystals remained unchallenged for many years. As far back as 1902, attempts to index the crystal faces of the naturally occurring crystals of the mineral calaverite (AuTe_2_), following the law of the simple rational indexes, had failed (Penfeld & Ford, 1902 ▶; Smith, 1903 ▶; Goldschmidt *et al.*, 1931 ▶). Indeed calaverite has incommensurate facets (Dam *et al.*, 1985 ▶; Dam & Janner, 1986 ▶) and exhibits in its diffraction pattern some extra peaks (called satellite reflections) that cannot be indexed using only three integer numbers, indicating the presence of a superstructure (Sueno *et al.*, 1979 ▶). In 1988, Schutte and de Boer solved the structure of a natural single crystal of calaverite using the superspace crystallography formalism and considering an incommensurate modulation attributed to the displacement of the Te atoms associated with the occupational modulation of the Au atoms (Schutte & de Boer, 1988 ▶).

In the 1960s, several examples of crystals presenting anomalies in physical properties related to the lack of three-dimensional periodicity were known (Daniel & Lipson, 1943 ▶; Hargreaves, 1951 ▶; Sato & Toth, 1961 ▶; Tanisaki, 1961 ▶, 1963 ▶; Jehanno & Perio, 1962 ▶) and the diffraction from periodic longitudinal and transversal distortions of crystals structure were well understood (Korekawa, 1967 ▶). In 1974, de Wolf presented an entirely new superspace description for the modulated phases in which the modulated structures were conveniently described through embedding into (3+*d*)-dimensional space. In this notation, the three represents the dimension of the physical three-dimensional space (*R*_3_) (in which the atoms are positioned, or in which the reciprocal space is observed), whereas the *d* represents the additional dimensions, orthogonal to *R*_3_, necessary to describe the modulation. The advantage of this description is the presentation of a three-dimensional modulated structure in superspace as a periodic one at the cost of higher dimensionality. This means that the concept of periodicity (translational symmetry) can be preserved (de Wolff, 1974 ▶). This work provided a common framework to interpret the observations on modulated crystals reported so far. It was understood that modulated crystals exhibit a periodic arrangement of their atoms, which is also superimposed by periodic deviations. However, the wavelength of the distortion and the vectors describing the translations of the network are not commensurate. In this approach, the real crystal is regarded as a three-dimensional section through the (3+*d*)-dimensional periodic ‘supercrystal’ and the diffraction pattern of the modulated crystal is regarded as the projection of its (3+*d*)-dimensional reciprocal lattice (Janner & Janssen, 1977 ▶; de Wolff, 1977 ▶; Janner & Janssen, 1980*a* ▶,*b* ▶; Janner *et al.*, 1979 ▶). After de Wolff’s original work, the definition of crystals evolved to ‘*ordered solids at a microscopic level presenting long-range order and conventional symmetry, formed by the regular arrangement of unit cells in all directions of the space*’.

The concept of crystals evolved once more after the discovery of quasicrystals by Daniel Shechtman in 1984 (Shechtman *et al.*, 1984 ▶) and according to the phenomenological approach taken by the International Union of Crystallography ‘*a crystal is any solid having an essentially discrete diffraction pattern*’.

Since these achievements, aperiodic crystallography has demonstrated tremendous progress, which would be impossible without developing adequate computational tools for solution, refinement, interpretation and visualization of the aperiodic structures.

Among the available methods to obtain the approximated structure of modulated crystals, common features were the determination of the average^1^ structure and the determination of approximated supercell structure. The average structure and the approximated supercell structure can be obtained by using standard direct or Patterson methods to solve the phase problem. While the average structure is obtained by neglecting the satellite reflections, the supercell structure is obtained by introducing many non-existing satellite reflections with zero intensity. Both approaches are likely to fail when modulation amplitudes are large or when intense satellite reflections of high order are present. In 2004, Oszlányi & Sütő proposed a direct method for structure solution, called *charge flipping*, based on the assumption that there are few regions within the crystal with high electron density (Oszlányi & Sütő, 2004 ▶). The *charge flipping* method does not directly imply atomicity and can be generalized to approach the phase problem in spaces of any dimension. Following this idea, Palatinus (2004 ▶) proposed an *ab initio* algorithm called *SUPERFLIP* for solving the modulated structures from X-ray diffraction data directly in superspace, without the intermediate step of determining the average or the approximated supercell structure, using simultaneously main and satellite reflection intensities (Palatinus & Chapuis, 2007 ▶). *SUPERFLIP* is nowadays a widespread tool for modulated structure solution.

In the 1980s, there were a few software packages available to deal with the refinement of modulated structures. Among them one can cite *REMOS* for single-crystal data and *PREMOS* for powder data (Yamamoto, 1982 ▶), *MSR* for single-crystal data (Paciorek & Uszynski, 1987 ▶; Paciorek, 1991 ▶) and *JANA* (Petricek *et al.*, 1985 ▶) for both single-crystal and powder diffraction data; the latest version of *JANA2006* is continuously being developed (Petříček *et al.*, 2014 ▶). *JANA2006* allows (i) data reduction and automatic identification of reflection conditions and superspace groups, (ii) structure solution directly in superspace using *SUPERFLIP* and (iii) refinement of nonmodulated, modulated and composite structures. Within *JANA2006*, one can carry out simultaneous refinement of atomic positional parameters, harmonic and anharmonic displacement parameters, molecular fragments and TLS formalism within the rigid-body approximation, including constraints for describing riding hydrogen atoms and modulation parameters described by different functions (harmonic, crenel, sawtooth as well as combined). *JANA2006* also allows the refinement of magnetic structures applying higher-dimensional magnetic superspace groups (Petříček *et al.*, 2010 ▶).

Most of the modulated compounds investigated to date are inorganic materials or metal alloys in which the atomic modulations (displacive or occupational) can be confined to electronic effects (van Smaalen, 2005 ▶; Janssen *et al.*, 2007 ▶) or could be interpreted in terms of frustration or competition, meaning that two or more mechanisms favour certain periodicities that are mutually incompatible (Yamamoto, 1982 ▶; Cummins, 1990 ▶; Aroyo *et al.*, 2006 ▶). There also exist a number of molecular modulated organic and organometallic compounds for which a detailed and precise description of the crystal structure (*i.e.* chemical properties, structural characteristics and modulation) provides information for the interpretation of the chemical stability and interactions between the molecules. Crystallographic information on many inorganic and organic modulated structures that is not usually included in the ‘conventional’ crystal structure databases is provided on the Bilbao Crystallographic Server (http://b-incstrdb.ehu.es/incstrdb/index.php) (Aroyo *et al.*, 2006 ▶).

## 3. Molecular modulated structures   

To the best of our knowledge, James and Saunder were the first to report on aperiodicity in organic molecular crystals (James & Saunder, 1947 ▶, 1948 ▶). In their reports, the structures of 4,4′-dinitrodiphenyl and halogenated diphenyl crystals were supposed to be built by layers of dinitrodiphenyl molecules in a face-centred array creating tubular cavities, also in a face-centred array in which the halogenated diphenyl molecules are placed. From analysis of the diffuse regular and the ‘ghost spectra’ lines observed in reciprocal space, the authors suggested that the regular spacing of the layers of the dinitrodiphenyl molecules, separated by 3.69 Å along the *c* axis, is modified by some periodic ‘error’ repeating itself at a distance of 12.3 Å, which coincides with the length of the halogenated diphenyl molecules. These structures were not refined following the IUCr standards (Chapuis *et al.*, 1997 ▶) and a detailed mechanism of the modulation has not yet been described.

**Figure 1:**
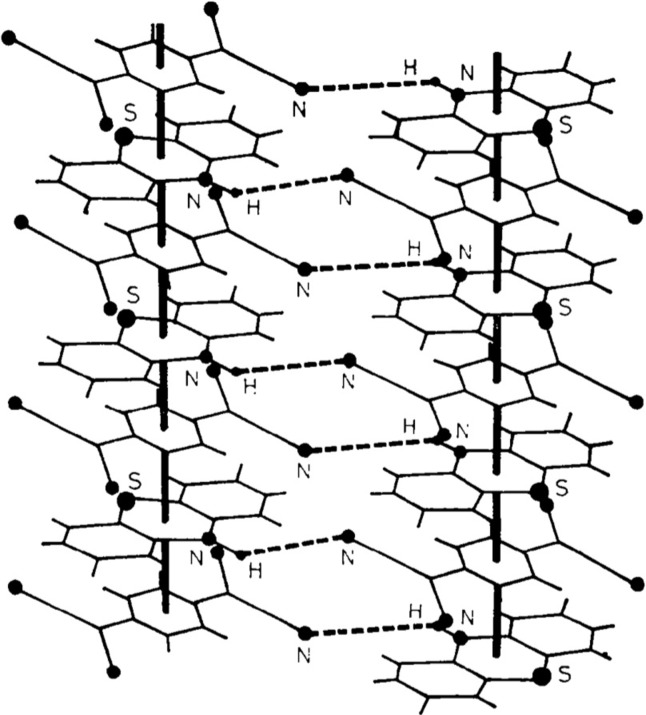
Representation of the π-stacking interaction between phenothiazine and 7,7,8,8-tetracyanoquinodimethane molecules within a column and a regular hydrogen-bond pattern presumed to be formed between molecules from adjacent columns. Reproduced with permission from Kobayashi (1974 ▶).

Up to the 1980s, most of the modulated structures have been solved and refined by means of a so-called atomic model which takes into account substitutional or displacive modulations of every atom in the crystal individually. In the atomic model, the position of an atom *j* (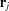) in the unit cell defined by the translation vector **n**, subject to a displacive modulation, is given by
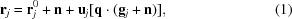
where 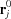 is the average position of the atom *j*, **u**_*j*_ is the periodic vector such that **u**_*j*_(*x*) = *u*_*j*_(*x* + 1), **q** is the modulation vector and the vector **g**_*j*_ defines the phase reference point of the displaced entity. In the atomic model **g**_*j*_ = 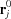 (de Wolff, 1977 ▶) or **g**_*j*_ = 0 (Kobayashi, 1974 ▶), while in a molecular displacement model **g**_*j*_ = *R*, where *R* is common for all atoms in a molecule or molecular segment. In general, in the atomic model approach all rigid-body molecular restraints and constraints are neglected. Nevertheless, the atomic model approach was successful in the determination of the structure of many minerals, alloys and inorganic modulated solids. The atomic model approach was also successfully used by Kobayashi (1974 ▶) to solve and refine the modulated structure of the molecular crystals of phenothiazine (PTZ) and 7,7,8,8-tetracyanoquinodimethane (TCNQ). The modulation observed in this structure was credited to the interplay between the short-range forces (hydrogen bonding, charge transfer and van der Waals interactions) and the long-range forces (dipole–dipole interaction between PTZ molecules). This interplay un­balances the charge-transfer interactions within a column of stacked PTZ–TCNQ molecules altering the regular hydrogen bond pattern likely to be formed between molecules from the adjacent columns (Fig. 1 ▶).

Despite the usefulness of the atomic model, it is not able to deal with the large and complex (high anharmonicity, discontinuous modulations) modulated structures. It should be stressed that in the molecular crystals, a translational or rotational displacement of an entire molecule, or one of its segments, is more likely because of the covalent/strong/directional interactions between the atoms. Besides, in any modulated structure of moderate size, restraints and constraints keep a chemically plausible geometry of the molecules during the refinement and prevent an excessive increase in the number of refined parameters. Finally, the modulation parameters are usually very sensitive to any deficiency in the basic structure model, thus any omission or inadequate description of a number of light atoms (usually hydrogen atoms) will significantly distort the final results.

Most of the organic molecular structures presenting incommensurately modulated phases described within the superspace approach show interplay between molecular conformation in a gas or liquid phase (which is assumed to be the ‘ideal’ conformation) and molecular conformation in the solid-state phase searching for a balance between better directional interactions and better solid-state packing. This can result in both displacive and occupational modulations of an entire molecule or group of atoms (Petricek *et al.*, 1985 ▶). The crystal-chemical analysis of modulated molecular compounds consists of the identification of the rigid fragments and their intra- and intermolecular interactions as well as the definition of the role played by the movable fragments or the functional groups, which have some degrees of freedom to rotate or tilt, in order to minimize or equilibrate steric effects. There are also cases of organic and organometallic molecular compounds presenting a thermotropic transition between nonmodulated and modulated phases for which knowledge of the geometry of the deviation from the underlying basic structure helps with the understanding of the mechanism of the transition.

**Figure 2:**
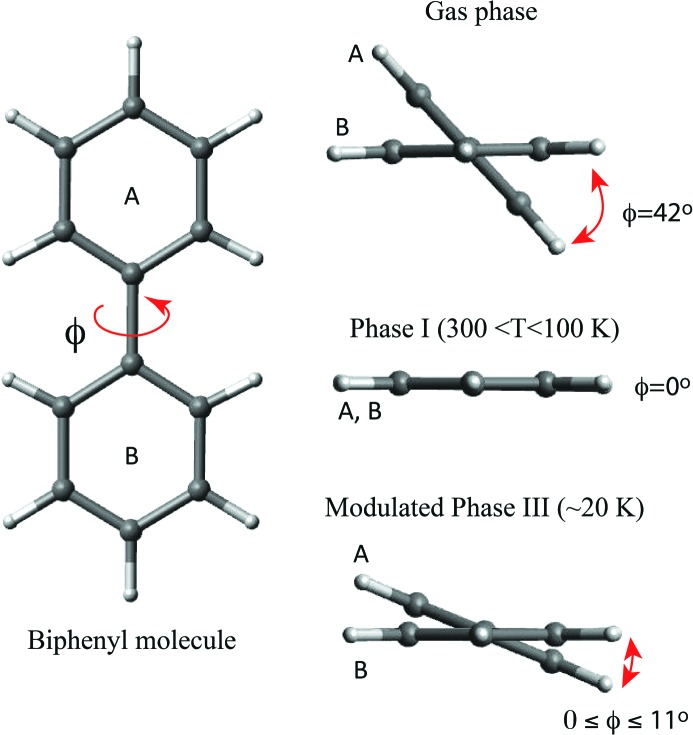
Biphenyl molecule and the representation of relative orientation of the rings in the different phases. The torsion angle ϕ between the rings is ∼42° in the gas phase and 0° in phase I (∼40 ≤ *T* ≤ 300 K). In the modulated phase III (*T* ≃ 20 K), the torsion angle between the rings varies harmonically between 0 ≤ ϕ ≤ 11°.

Biphenyl (C_12_H_10_) is the classic example of a hydrogen-rich molecular compound presenting modulated incommensurate crystalline phases. Owing to the repulsion of the *ortho*-hydrogen atoms, the conformation of the molecule in the gas phase is nonplanar and the torsion angle ϕ between the planes of the two phenyl rings along the central single C—C bond is around 42°. From room temperature down to ∼45 K (phase I), the biphenyl crystal structure is described in the space group *P*2_1_/*a*, with two planar molecules and a time-averaged ϕ of 0°; the molecules are related by an inversion centre (Hargreaves & Rizvi, 1962 ▶; Lenstra *et al.*, 1994 ▶). The deuterated biphenyl structure at *T* ≃ 20 K (phase III) was initially solved using a commensurate approach (Cailleau *et al.*, 1979 ▶) and further refined using the superspace approach in the space group *Pa*(0β0)^2^ (Baudour & Sanquer, 1983 ▶). The structure refinement was based on a harmonic displacive modulation and rigid-body restraints for the positional parameters of the phenyl atoms. The most remarkable feature of the modulation observed in biphenyl is the incommensurate change of the torsion angle ϕ with an amplitude of ∼5.5° for each ring (maximum relative torsion of ϕ ∼ 11°) (Fig. 2 ▶). The modulation in biphenyl is explained on the assumption that in phase I, the intermolecular packing forces and the intramolecular H⋯H steric repulsion forces that compete to define the molecular geometry seem to be of the same order of magnitude leading to a planar molecule, whereas in phase III the imbalance between these two forces defines the molecular conformation leading to the observed modulation. The force imbalance is related to the enmeshed rotations between phenyl rings of nearest neighbours molecules (Heine & Price, 1985 ▶).

In most of the modulated molecular structures, which were successfully determined using the superspace approach, rigid molecular fragments were identified and refined subject to rigid-body restraints or constraints. In general, the modulation does not seem to affect the internal geometry of the rigid molecular fragments (defined by the covalent bonds) but rather affects their relative positions and orientations. Structural analysis in which the atoms in these rigid fragments were freely refined presented usually better *R* values, most likely owing to the significant increase in the number of refined parameters, without altering the overall description of the structures.

In the following sections, we will highlight some examples of organic and organometallic molecular complexes presenting modulated phases as well as comment on the plausible origin of the observed modulation. Obviously, the list of selected examples is biased and for illustration only and aims to continue previous efforts to categorize modulated molecular structures (Schönleber *et al.*, 2014 ▶). More comprehensive work on this subject still requires larger and better experimental data sets highlighting the strength and the packing role of hydrogen bonds (including its weakest variant), C—H⋯π interactions, van der Waals interactions and dipole−dipole interactions and halogen bonds (Desiraju, 2013 ▶).

### 3.1. Hexamethylenetetramine and aliphatic acids: modulation and hydrogen bonds   

**Figure 3:**
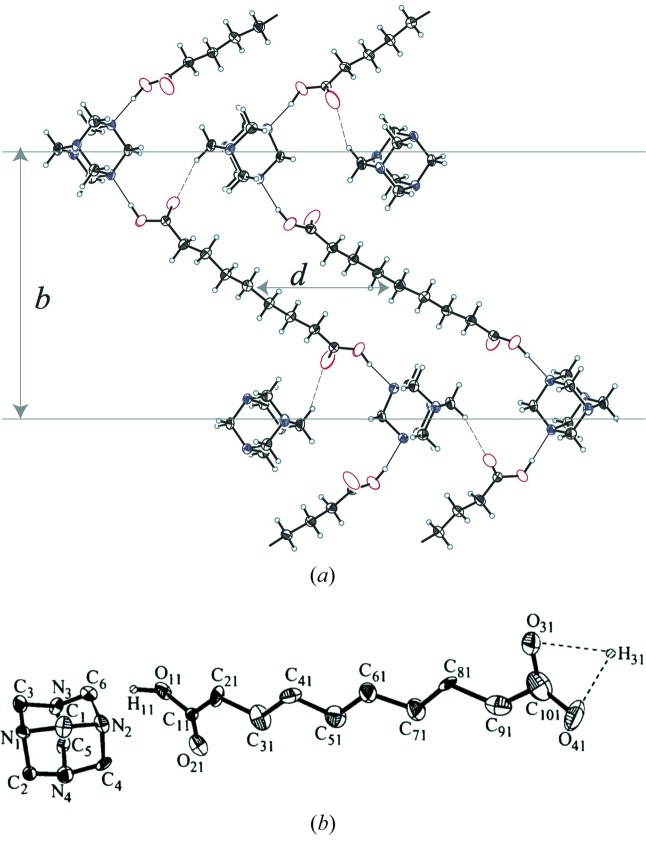
(*a*) Overall structure packing of the HMT–C*n* crystal indicating layers of pure HMT stacked over layers of pure C*n* aliphatic chains. The arrows marked *b* and *d* indicate the different HMT–C*n* crystals. (*b*) The HMT–C10 structure showing the delocalization of the H atoms at one end of the aliphatic chain. Picture taken from Bussien Gaillard *et al.* (1998 ▶).

The 1:1 co-crystals of hexamethylenetetramine N_4_(CH_2_)_6_ and aliphatic dicarboxylic acids HOOC(CH_2_)_*n*−2_COOH are denoted HMT–C*n*. Complexes with 5 ≤ *n* ≤ 13 form lamellar structures in which the layers of pure HMT stack over layers of pure C*n* (Fig. 3 ▶). The stabilization of the adducts is essentially ensured by the C=O—H⋯N hydrogen bonds connecting the HMT and C*n* moieties restrained by the C—H⋯O—C hydrogen bonds between the terminal acid function and a carbon atom of a neighbouring hexamethylenetetramine and van der Waals interactions between C*n* chains. Depending on the number of C atoms (*n*) in the aliphatic chain, HMT–C*n* co-crystals exhibit ordered, disordered, twinned as well as modulated phases upon cooling from ∼350 K down to 80 K (Hostettler *et al.*, 1999 ▶; Gardon *et al.*, 2001 ▶; Bonin *et al.*, 2003 ▶; Pinheiro *et al.*, 2003 ▶). It has been reported that most of the structural phase transitions are reversible except the one for HMT–C9 at 240 K (Hostettler *et al.*, 1999 ▶). Bussien Gaillard *et al.* (1996 ▶, 1998 ▶) showed that the RT structures of both HMT–C8 (hexamethylenetetramine suberate) and HMT–C10 (hexamethylenetetramine sebacate) adducts are strongly modulated and described by the space group *P*2_1_(α0γ). The structures consist of alternating sheets of hexamethylene­tetramine and aliphatic acid chains connected by the C=O—H⋯N hydrogen bonds. Each layer of aliphatic acid chain shows alternating areas, in which the zigzag planes and the chain axes of the aliphatic chains are roughly parallel. In the adjacent areas, the zigzag planes are rotated by ∼65°. For both adducts, the C=O—H⋯N hydrogen bonds at opposite ends of the dicarboxylic acid chains are distinguishable. Whereas a well defined C=O—H⋯N hydrogen bond, defining a carboxylic group, was confirmed at one chain end, a delocalization of the H atom, defining a carboxylate group, was observed at the other end (Fig. 3 ▶). The packing of aliphatic chains with the localized and delocalized H atoms are incompatible and were supposed to be responsible for the incommensurability of the structure. In both HMT–C8 and HMT–C10, the modulation does not affect the overall geometrical molecular features of the HMT molecules and the C*n* chains that were refined subject to molecular restraints.

### 3.2. 2-Phenylbenzimidazole and adamantan-1-ammonium 4-fluorobenzoate: modulation and molecular conformation constrained by hydrogen bonds   

**Figure 4:**
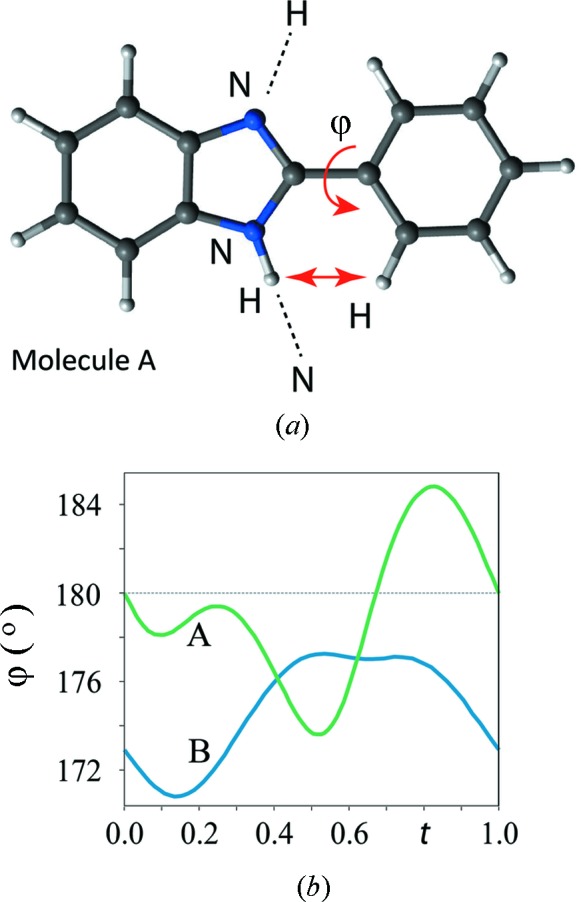
(*a*) 2-Phenylbenzimidazole molecule with indication of the intramolecular contacts and torsion angle ϕ. (*b*) Modulation plot of the torsion angle ϕ between the two planar moieties as indicated in (*a*) for molecule A (green) with an amplitude of ±5° and molecule B (blue) with an amplitude of ±3°. The grey line indicates the value ϕ = 180° corresponding to a flat molecule. Figure taken from Schönleber (2011 ▶). Varying *t* for a certain geometric parameter, like a rotation angle, from *t* = 0 to *t* = 1, gives all values for this parameter occurring anywhere in the three-dimensional aperiodic crystal structure along the physical space *R*_3_ (Janssen *et al.*, 2006 ▶).

The 2-phenylbenzimidazole (C_13_H_10_N_2_) molecule is formed by a planar phenyl ring and a planar benzimidazole moiety (Fig. 4 ▶ *a*). From room temperature down to 90 K the crystals exhibit a disordered incommensurately modulated structure described by the space group *C*2/*c*(0β0)*s*0 (Zuñiga *et al.*, 2006 ▶). Each of the eight molecules in the unit cell can occupy two configurations related by an inversion centre. The calculated torsion angle ϕ between the phenyl ring and the benzimidazole in the gas phase is about 160° (Catalán *et al.*, 1997 ▶). In the modulated phase, ϕ varies along the crystal as shown in Fig. 4 ▶(*b*). The modulation in the torsion angle seems to result from the balance between the steric effects between the *ortho*-hydrogen atoms pushing towards large ϕ angles observed in the gas phase and the N—H⋯N (average *d*_H⋯N_ ≃ 1.82 Å) hydrogen bond networks that connect molecules in the solid-state phase pushing towards small ϕ angles.

**Figure 5:**
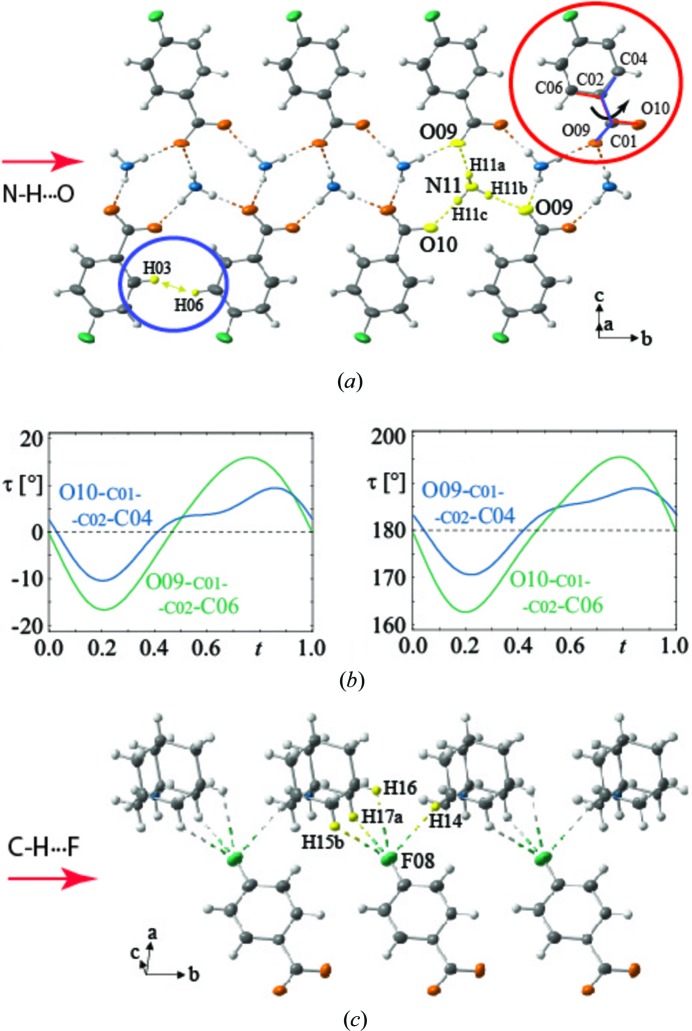
Crystal structure of adamantan-1-ammonium 4-fluorobenzoate indicating (*a*) the network of N—H⋯O bonds as well as the steric effects between hydrogen atoms of the neighbouring molecules and (*b*) the torsion angles between atoms of the fluorophenyl group and the carboxylate group. (*c*) The C—H⋯F hydrogen bonds. Figures adapted from the work of Schönleber *et al.* (2014 ▶).

The organic salt compound formed by one proton transference from the 4-fluorobenzoate (C_7_H_4_FO_2_^−^) anion towards the adamantan-1-ammonium (C_10_H_18_N^+^) cation presents an incommensurately modulated phase at low temperature (Schönleber *et al.*, 2014 ▶). Its structure refinement was carried out in the monoclinic space group *P*2_1_/*n*(α0γ)00. The C—O bond lengths in the carboxylate group were found to be similar [*d*_av_ ≃ 1.258 (4) Å] and the crystal packing is assured by a network of strong N—H⋯O and weak C—H⋯F hydrogen bonds (Figs. 5 ▶ *a* and 5*c*). Despite the large amplitude of the harmonic modulation the adamantine cage, the fluoro­phenyl group and the carboxylate group were found to be rigid. Besides, the plane of the carboxylate group was found to rotate periodically with a maximum amplitude of ∼12° with respect to the plane of the fluorophenyl ring (Fig. 5 ▶ *c*). The interplay between the optimal conformation and dense packing of the molecular ions and the steric effects between hydrogen atoms of the neighbouring phenyl rings explain the modulation.

### 3.3. C_6_H_4_S_2_AsCl: modulation, steric effects and electron-rich atomic contacts   

**Figure 6:**
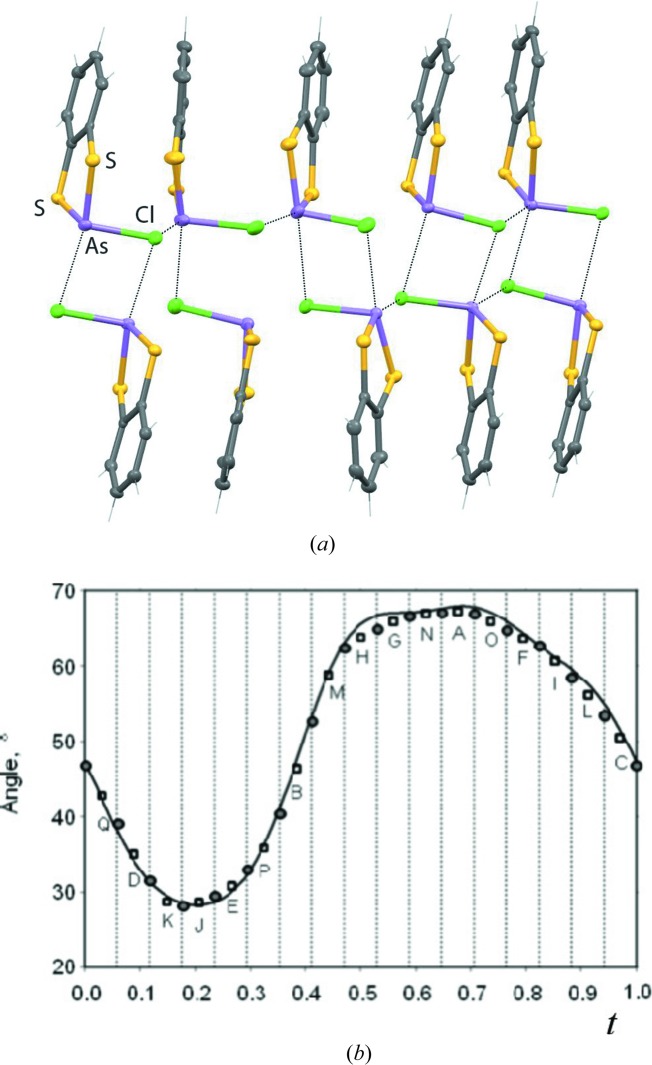
(*a*) Superstructure (*Z*′ = 17) approximation of the C_6_H_4_S_2_AsCl structure showing the dimer and ribbons as well as the relevant As⋯Cl contacts. (*b*) The angle between the S_2_C_6_ planes and the (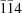)_17_ direction for the incommensurate refinement (line) and in the commensurate approximation (circles and squares) taken from the work of Bakus II (2013 ▶).

Organometallic compounds 2-chlorobenzo-1,3,2-dithiastibole (C_6_H_4_S_2_SbCl) and 2-chlorobenzo-1,3,2-dithiarsole (C_6_H_4_S_2_AsCl) are isostructural molecules which present quite different crystalline structures. The first is described in the orthorhombic space group *Pbca* (Shaikh *et al.*, 2006 ▶) while the latter is described in the triclinic superspace group *P* 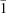
(αβγ) (Bakus II *et al.*, 2013 ▶). The C_6_H_4_S_2_AsCl molecules are packed in dimers by short As⋯Cl contacts and in ribbons by longer As⋯Cl contacts leading to short contacts between the dithiolate (C_6_H_4_S_2_) groups. The observed arrangement of the ribbon columns allows the space to be densely filled with unfavourable inter-ribbon interactions between the dithiolate groups. The steric effects between the dithiolate groups are minimized by a periodic change in the entire C_6_H_4_S_2_AsCl molecule orientation followed by shifts along the ribbon axis to relieve the short contacts within the ribbons as represented by the superstructure (*Z*′ = 17) approximation shown in Fig. 6 ▶. As the As⋯Cl/S contacts are ∼0.2 Å shorter than their equivalent Sb⋯Cl/S contacts, there would be no space to accommodate the C_6_ aromatic rings in the packing similar to that observed in C_6_H_4_S_2_SbCl.

### 3.4. Modulation in urea host–guest compounds   

**Figure 7:**
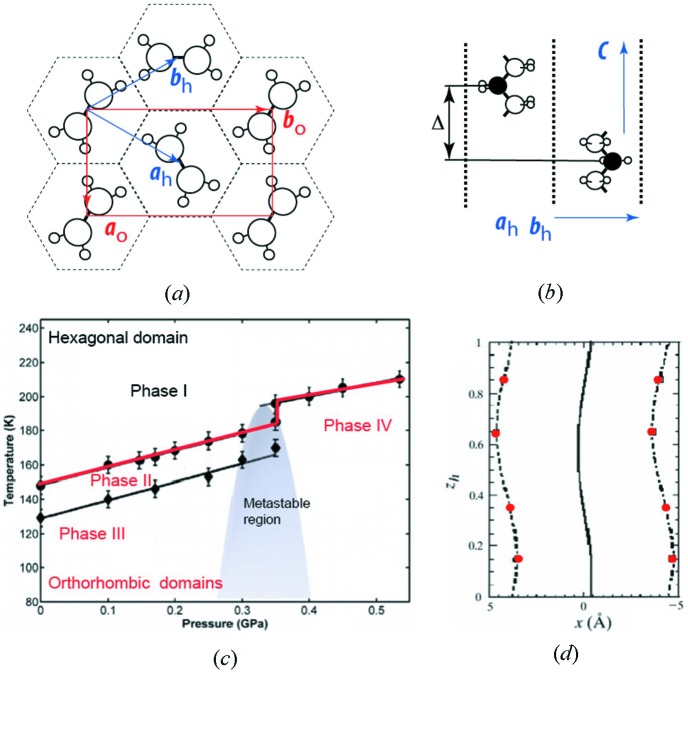
(*a*, *b*) Schematic herringbone-like arrangements showing the translational and orientational disorder of the *n*-alkane molecules inside the parallel channels built by the urea host hexagonal sublattice. Hexagonal (h) and orthorhombic (o) lattice parameters used to describe the structures in different phases are shown. (*c*) Phase diagram (*P*, *T*) of the fully deuterated *n*-nonadecane/urea inclusion compound [adapted from Toudic *et al.* (2011 ▶)]. (*d*) *x*–*z*_h_ plot of the refined modulation function (solid line) together with the positions of the N (circles) atoms in two opposite tunnel walls perpendicular to the octane molecular plane. The dashed lines are the sinusoidal modulation functions fitted to the N positions. Adapted from the work of Peral *et al.* (2001 ▶).

In the urea inclusion compounds, long-chain alkane or substituted alkane *guest* molecules are densely packed within one-dimensional *host* tunnels, formed by a honeycomb-like hydrogen-bonded network of urea molecules as shown in Figs. 7 ▶(*a*) and 7(*b*) (Smith, 1952 ▶). In general, guest structures are dynamically disordered owing to the random possible orientation of the molecules within the host tunnels as indicated by strong diffuse scattering (Forst *et al.*, 1987 ▶). Empty urea tunnel structures are unstable and the removal of the guest molecules from urea inclusion compounds leads to the instantaneous collapse of the structure to form a pure urea higher density structure. Depending on the ratio *c*_host_/*c*_guest_ between the lengths of the host and guest lattice axes along the tunnels, also known as a misfit parameter, the urea inclusion crystals can be considered as either commensurately or incommensurately modulated (Harris & Thomas, 1990 ▶). The aperiodicity resulting from different interacting length scales seems to play a major role and controls the properties like crystal growth, guest ordering, absorption and guest-exchange processes (Harris, 2007 ▶). During the cooling, most of the urea inclusion compounds undergo phase transitions into less symmetric structures, which normally involve a volume multiplication of the host structure and a partial ordering of the guest molecules.

Most of the urea inclusion compounds show no detectable intermodulation between host and guest substructures. Thus, strong Bragg peaks associated with the different intercalated subsystems can be measured but no satellite intensities can be observed. The *n*-nonadecane–urea inclusion compound was the first in which intermodulations were observed in both the host and guest substructures (Lefort *et al.*, 1996 ▶; Weber *et al.*, 1996 ▶). The evolution of the structure as a function of temperature and pressure reveals a rich sequence of phase transitions (Fig. 7 ▶ *c*), where hexagonal to orthorhombic transitions are mediated by the displacive intermodulation between the host–guest structures followed by a change in the superspace group symmetry (Toudic *et al.*, 2008 ▶, 2011 ▶; Mariette *et al.*, 2013 ▶).

The structures of the inclusion compound crystals of *n*-heptadecane–urea (Weber *et al.*, 1997 ▶) and of *n*-octane–urea (Peral *et al.*, 2001 ▶) were refined using the superspace approach in order to understand the host and guest mutual intermodulation effects. The symmetry of both compounds was described by the space group *P*6_1_22(00γ), despite the violation of the 6_1_ screw axis extinction rule by the guest modulated structure. The modulation of the host structures was found to be very weak, whereas the modulation of the guest structure was interpreted by an adaptation of the guest molecules to the host structure. The origin of the modulation was attributed to the distribution of the N atoms (NH_2_ groups), which are located at two different distances on the tunnel axis within each urea tunnel wall. The modulation of the guest molecules has maximum amplitude when the CH_2_ groups of the *n*-heptadecane and *n*-octane molecules are facing the channel walls and follow the tunnel modulation in such a way that the distances between their centres and the N atoms belonging to the walls perpendicular to the molecular plane are almost equal (Fig. 7 ▶ *d*).

### 3.5. Commensurate approach: useful but not often used   

In the examples above, we made it clear that the use of the superspace approach to describe aperiodic structures goes far beyond the simple reduction of the number of refined parameters. Indeed, the superspace description reveals the real symmetry (as opposed to the crystallographic averaged) and the real structure of the crystals. Nevertheless, there are cases in which the use of superspace also provides the simplest description of the relationship between the modulated and nonmodulated phases. This can be particularly useful for structures with complex incommensurately modulated phases and/or with multiple molecules in the crystal asymmetric unit (*Z*′ > 1). High-*Z*′ structures are known to present some structural complexity arising from the differences in the kinetics and the thermodynamic evolution during the aggregation/nucleation and crystal growth processes (Steed, 2003 ▶; Desiraju, 2007 ▶) and are often characterized by pseudosymmetry or by modulation and can be a distorted or a modulated version of the lower *Z*′ structures (Bernstein *et al.*, 2008 ▶). The next section presents cases where the superspace commensurate approach was used to describe the molecular organic structures both due to their complexity or due to high *Z*′.

#### 3.5.1. *p*-Chlorobenzamide   

**Figure 8:**
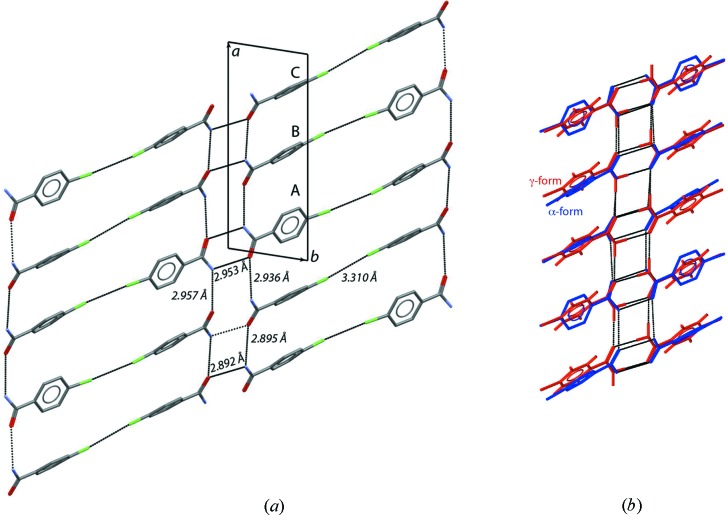
(*a*) Crystal structure of *p*-chlorobenzamide in the α-form (*T* < 317 K) indicating the three independent molecules in the asymmetric unit (*Z*′ = 3), as well as the intermolecular hydrogen and halogen bonds. (*b*) The α-form (blue) and the γ-form with *Z*′ = 1 (red) structure superposition

The room-temperature structure (*α*-form) of the organic compound *p*-chlorobenzamide, C_7_H_6_ClNO, was initially described in the space group *P* 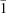 with three independent molecules (A, B and C ) in the asymmetric unit, *Z*′ = 3 (Fig. 8 ▶) (Taniguchi *et al.*, 1978 ▶). The crystal structure is composed of one centrosymmetric (A–A) and two asymmetric (B–C and C–B) dimer units. Dimers are linked by symmetric N—H⋯O=C hydrogen bonds between amide groups to form endless chains interconnected by Cl⋯Cl halogen bonds (Fig. 8 ▶). Molecules B and C are approximately parallel to each other, whereas the plane of the benzene ring for molecule A at room temperature makes angles of ∼34.0 and ∼37.8° (∼40.0 and ∼44.4° at 150 K) with those for B and C, respectively. The torsion angles ϕ between the plane defined by the phenyl ring and the one defined by the amide group are 19.0, 33.9 and 28.6° for molecules A, B and C, respectively. At 317 K, *p*-chloro­benzamide undergoes a solid-state phase transition to a γ-form described in the space group *P* 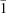 with only one independent molecule in the asymmetric unit, *Z*′ = 1, with ϕ = 27.3°. In the γ-form the intramolecular torsion angle ϕ = 27.3 (3)° is the same as the mean value [ϕ_ave_ = 27 (5)°] of the corresponding torsion angles of the three independent molecules of the α-form (Takaki *et al.*, 1978 ▶). Schönleber *et al.* (2003 ▶) reinvestigated the structure of *p*-chlorobenzamide using the superspace approach to deduce a simple relation between the α-form and γ-form. In this approach, both phases present *Z*′ = 1 and the same bonding scheme between the molecules. The α-form was refined considering a commensurate modulation in the space group *P* 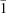
(αβγ) with three different orientations of the *p*-chloro­benzamide molecules recovered from the structure refinement by selecting the three specific values of the modulation parameter *t*. The description of the *p*-chlorobenzamide structure using the same reference structural model within the superspace approach leads to a simple description of the supramolecular synthon frustration during the γ → α form phase transition providing a unified picture of the relationship between the modulated (high-*Z*′) and nonmodulated (low-*Z*′) phases.

#### 3.5.2. Metal complexes and (15-crown-5)   

**Figure 9:**
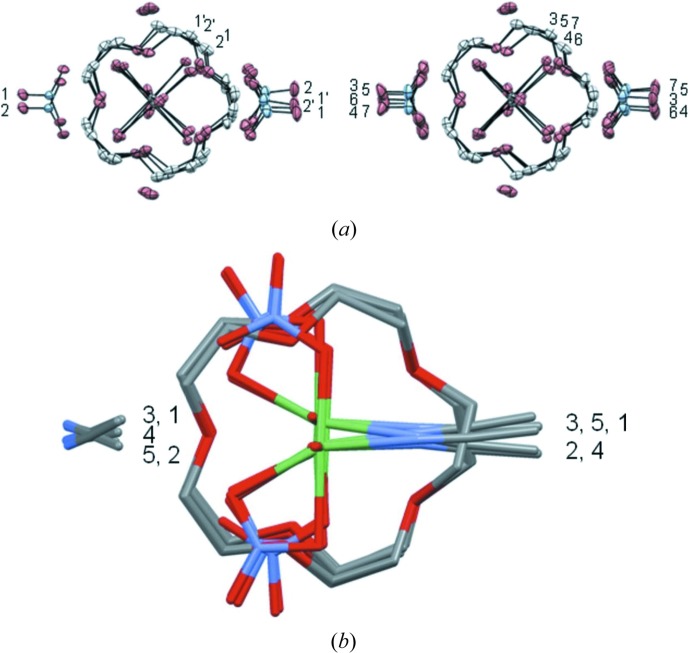
(*a*) Projection of the seven independent molecules in [Ni(H_2_O)_6_](NO_3_)_2_](15-crown-5)·2H_2_O at *T* = 90 K showing the two basic positions of the cation and the 15-crown-5 groups as well as the multiple positions of the nitrate ions. (*b*) Projection of the five independent molecules of [Ni(MeCN)(H_2_O)_2_(NO_3_)_2_](15-crown-5)·MeCN at *T* = 90 K showing the two basic orientations of the nickel. Pictures taken from Siegler *et al.* (2011 ▶, 2012 ▶).

The structural family of the organometallic complexes [*M*(H_2_O)_2_(15-crown-5)](NO_3_)_2_ (*M* = Mg, Al, Mn, Cr, Fe, Co, Ni, Cu, Zn, Pd) has been investigated and classified according to the dimensionality of their hydrogen-bond network (Hao, Parkin & Brock, 2005 ▶; Hao, Siegler *et al.*, 2005 ▶; Siegler *et al.*, 2008 ▶, 2010 ▶). These investigations led to the discovery of the complexes of [Ni(H_2_O)_6_](NO_3_)_2_](15-crown-5)·2H_2_O and [Ni(MeCN)(H_2_O)_2_(NO_3_)_2_](15-crown-5)·MeCN which present a rich sequence of structural phase transitions characterized by the ordering of the NO_3_ and Ni complexes upon cooling (Siegler *et al.*, 2011 ▶, 2012 ▶). The crystal packing of these two Ni compounds upon cooling and their structures are highly disordered at room and intermediate temperatures but fully ordered at low temperature. In the ordering process both complexes present one incommensurate modulated phase. The modulated phases were refined in a supercell with *Z*′ = 7 for the aqua and *Z*′ = 5 for MeCN complexes, using a rather complicated refinement strategy and significant increase in the number of refined parameters (Fig. 9 ▶). The description of the modulated phases as well as their role in the ordering of the molecules still need further investigation.

It is worth mentioning that the room-temperature structures of the two polymorphic forms of the compound named diaqua(15-crown-5)copper(II) dinitrate, [Cu(H_2_O)(15-crown-5)](NO_3_)_2_, similar to previous ones, have already been investigated using the superspace approach (Schönleber & Chapuis, 2004 ▶). In this work, the small *Z*′ = 1/2 disordered structure (Dejehet *et al.*, 1987 ▶) was chosen as the reference model for the larger *Z*′ = 10 polymorph and was further refined in the space group *Pc*(α0γ)*s*. The *Z*′ = 10 polymorph was successfully described with the crown complexes presenting two different orientations with occupation described by complementary crenel functions providing, once again, a unified description of the polymorphs.

## 4. Modulated oxide materials   

**Figure 10:**
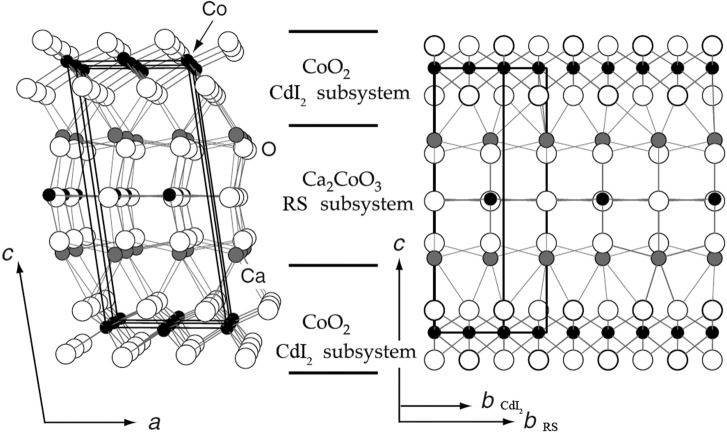
The misfit crystal structure of [Ca_2_CoO_3_]_0.62_CoO_2_. The CdI_2_ and rock-salt (RS) subsystems are denoted. Displacement modulations are not shown for clarity. The figure is adapted from Miyazaki *et al.* (2002 ▶).

Rational design of solid-state materials with new functionalities requires a comprehensive understanding of the relationships between the crystal structure and the functional properties. The macroscopic behaviour of many materials largely depends on the nature and the concentration of point or extended defects and their mutual interaction. Association of the defects owing to intersite repulsive or attractive forces coupled with the lattice strain can lead to complex ordering patterns that are strikingly different from an averaged picture based on random distributions of defect species over the relevant sublattices. These patterns are often driven by a tendency to minimize the free energy of the system through adopting a so-called uniform ordering, where the ordered species or structure fragments are distributed as homogeneously as possible (Perez-Mato *et al.*, 1999 ▶; González *et al.*, 2011 ▶, 2012 ▶). Another source of structural complexity originates from co-existing competing distortion modes favouring a frustrated structure, where an incommensurately modulated state provides a reasonable compromise satisfying both distortions simultaneously. The distortion can be related to a cooperative framework deformation optimizing the chemical bonding for a certain set of atoms, atomic displacements of the lone pair cations, off-centre polar displacements and other factors. Incommensurability can also be confined to electronic instabilities, manifesting itself as charge- and spin-density waves and in orbital ordering. Incommensurate modulations are intrinsic in many modular structures, where the continuous structure blocks with different sublattices are mutually modulated with a periodicity to be a common multiple of the periodicites of the constituting blocks. Well known examples of such misfit homologous series are provided by the thermoelectric cobaltites [(*M*O)_*n*_]_*x*_(CoO_2_), where two subsystems are formed by the alternating CoO_2_ octahedral layers with the ‘CdI_2_’-type structure and the rock-salt-type (*M*O)_*n*_ blocks (Miyazaki *et al.*, 2002 ▶, 2003 ▶; Valkeapää *et al.*, 2007 ▶; Shizuya *et al.*, 2007 ▶) and superconducting [(*A*S)_*n*_]_1+*x*_(*M*S_2_)_*m*_ sulfides with the intergrowth of the NbS_2_-type and the rock-salt-type AS blocks (Wiegers, 1996 ▶; Meerschaut, 1996 ▶, and references therein) (Fig. 10 ▶).

Owing to the diverse crystal chemistry and crystal physics behind numerous families of modulated crystals, there is not much sense looking for the unified origins and the mechanisms of the aperiodic order. Moreover, linking the peculiarities of the incommensurate order with the functional properties of the materials would require individual consideration in every particular case. However, there should be no doubt that such links must be established, as they can be decisive for the behaviour of the material. We will try to illustrate these links using several examples of oxide materials with modulated structures of different nature.

### 4.1. Modulations in the anion sublattice   

**Figure 11:**
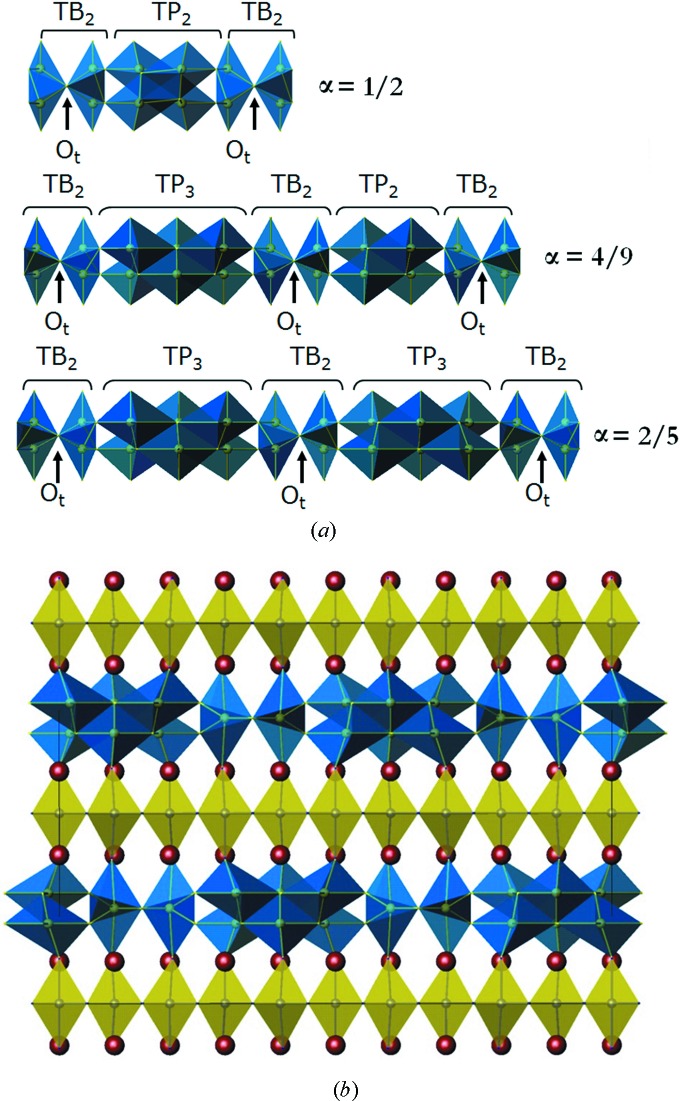
(*a*) Sequences of FeO_5_ polyhedra in (Fe_2_O_2+δ_) rock-salt blocks of Sr_4_Fe_6_O_12+δ_ phases with different α components of the modulation vector. Oxygen atoms in the tetrahedral interstices are denoted as O_t_. Trigonal bipyramids and tetragonal pyramids FeO_5_ are denoted TB and TP, respectively. (*b*) Crystal structure of Sr_4_Fe_6_O_12.8_. FeO_6_ octahedra are yellow, FeO_5_ polyhedra are blue and Sr atoms are shown as brown spheres.

Perhaps, a quest for high-temperature superconductivity in layered cuprates in the last decades of the 20th century has raised the attention of solid-state chemists and material scientists to the aperiodic order because one of the central family of the superconducting compounds, the Bi-based Bi_2_Sr_2_Ca_*n*−1_Cu_*n*_O_2*n*+4+δ_ cuprates, appeared to be incommensurately modulated (Gao *et al.*, 1988 ▶, 1989 ▶; Petricek *et al.*, 1990 ▶; Yamamoto *et al.*, 1990 ▶; Mironov *et al.*, 1994 ▶). In these phases, represented as an intergrowth of the perovskite and rock-salt structure blocks, the dimensions across the stacking axis are given by the Cu—O distances of the CuO_2_ perovskite-type layers, which fall in the range 1.9–1.95 Å. In order to match the perovskite block geometrically, the Bi—O interatomic distances in the adjacent rock-salt (Bi_2_O_2+δ_) block should be in the range 2.7–2.75 Å, which is too long for the relatively small Bi^3+^ cation. In order to mitigate the mismatch, the rock-salt block undergoes cooperative atomic displacements, which reduce the Bi—O bond lengths and restore a coordination number typical for this cation. The resulting incommensurate displacive modulation is coupled with a concomitant occupational modulation owing to the introduction of an interstitial (δ) oxygen atom. This over-stiochiometric oxygen provides the hole doping to the CuO_2_ layers necessary for a transition to the superconducting state. At the same time, the cooperative atomic displacements in the (Bi_2_O_2+δ_) blocks induce distortions in the CuO_2_ layer lowering the superconducting transition temperature (*T*_c_), which is particularly sensitive to the geometry of these layers. Increasing the thickness of the perovskite block on going from the first member of the Bi_2_Sr_2_Ca_*n*−1_Cu_*n*_O_2*n*+4+δ_ series to the members with *n* = 2 or 3 raises *T*_c_ from ∼20 K to ∼86–108 K as the internal CuO_2_ layers of the thicker perovskite blocks are less affected by the geometric distortions. In another member of this intergrowth family, Sr_4_Fe_6_O_12+δ_ (*n* = 1, Fe_2_Sr_2_FeO_6+δ/2_), the incommensurate modulation within the (Fe_2_O_2+δ_) rock-salt block occurs in a similar manner. An insertion of the interstitial oxygen atoms with the periodicity given by the modulation vector **q** = αa* and oxygen nonstoichiometry index δ = 2α is coupled with the atomic displacements optimizing the Fe—O distances and the Fe coordination environment, which fluctuates between tetragonal pyramid and trigonal bipyramid (Rossell *et al.*, 2004 ▶, 2005 ▶; Abakumov *et al.*, 2008 ▶; Mellenne *et al.*, 2004 ▶; Pérez *et al.*, 2006 ▶) (Fig. 11 ▶).

The Co-doped derivatives of the Sr_4_Fe_6_O_13_ phase have been considered as promising mixed ionic electronic conductors. However, high ionic conductivity in these materials has been demonstrated to occur due to exsolution of the SrFe_1−*x*_Co_*x*_O_3−δ_ perovskite (Fossdal, 2001 ▶; Tarancón *et al.*, 2010 ▶). The undoped Sr_4_Fe_6_O_13_ compound shows only very minor oxygen ionic conductivity of ∼6 × 10^−4^ and ∼3 × 10^−4^ Scm^−1^ for partial oxygen pressures *p*(O_2_) = 10^−1^–10^5^ Pa at 1173 K in a bulk form and negligible conductivity (beyond the measurable limit) in thin films (Avdeev *et al.*, 2002 ▶; Solís *et al.*, 2010 ▶). This is in drastic contrast to other perovskite-based ferrite Sr_2_Fe_2_O_5_ with the brownmillerite-type structure, where the oxygen-ion conductivity is at least two orders of magnitude higher. Although a wide range of oxygen nonstoichiometry has been reported for Sr_4_Fe_6_O_12+δ_ (Avdeev *et al.*, 2002 ▶; Solís *et al.*, 2010 ▶), the interstitial oxygen atoms remain immobilized in the modulated (Fe_2_O_2+δ_) rock-salt block due to strong coupling between their ordering and local strain that causes aperiodic compressive deformation of the (Fe_2_O_2+δ_) blocks. Additional detrimental effect on oxygen ion conductivity in this material originates from the abundant antiphase shifts within the (Fe_2_O_2+δ_) blocks which cut off the diffusion pathways (Rossell *et al.*, 2013 ▶).

**Figure 12:**
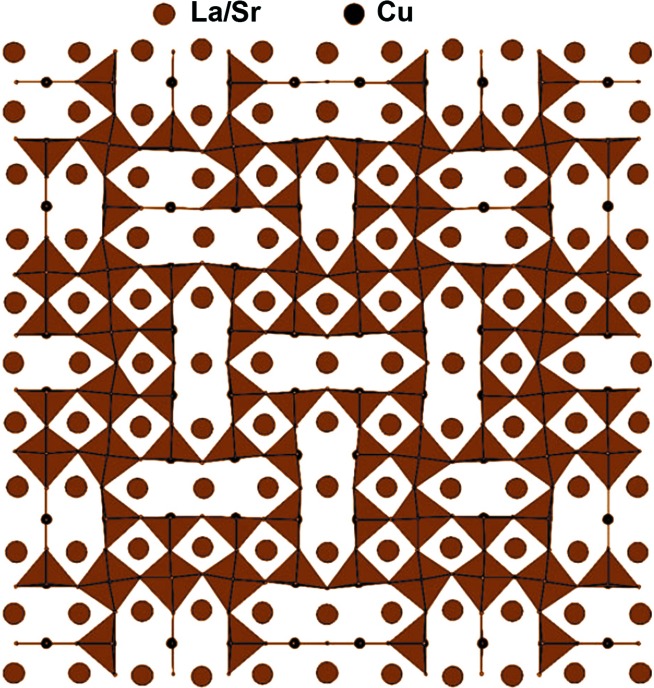
Ordering pattern of oxygen atoms and vacancies in the CuO_1.5_ perovskite layer of the LaSrCuO_3.5_ modulated structure. The figure is adapted from Hadermann *et al.* (2007 ▶).

The oxygen deficiency in the perovskite–rock-salt intergrowths can cause incommensurate ordering within the perovskite blocks, as exemplified by anion-deficient Ruddlesden–Popper structures *A*_*n*+1_ *B*_*n*_O_3*n*+1_. In the potential cathode material for solid oxide fuel cells, LaSrCuO_3.5+δ_, the oxygen vacancies are concentrated in the CuO_1.5+δ_ perovskite layers (Mazo *et al.*, 2007 ▶; Hadermann *et al.*, 2007 ▶). The oxygen vacancies are ordered in a complex fashion creating an incommensurately modulated pattern of CuO_4_ squares, CuO_5_ tetragonal pyramids and CuO_6_ octahedra (Fig. 12 ▶). Such ordering is governed by the Jahn–Teller effect intrinsic in the Cu^2+^ (*d* ^9^) cations, which promotes the removal of the oxygen atoms from the CuO_6_ octahedra reducing the Cu coordination down to tetragonal pyramidal and square planar. Heating easily suppresses this ordering causing high oxygen mobility in this compound at elevated temperatures (Savvin *et al.*, 2005 ▶).

It is worth noting that the family of perovskite–rock-salt intergrowths can be significantly expanded through a specific shearing mechanism resulting in the interruption of the perovskite and rock-salt layers and connecting them to each other. In contrast to the layered perovskite–rock-salt intergrowths, the stacking of the perovskite and rock-salt blocks can occur along two directions resulting in so-called ‘stair-like’ incommensurate structures with the common formula Bi_4*z*_Sr_6*z*_Fe_1−10*z*_O_4*z*+1_. The building principles and crystal structure of these intergrowth materials are understood with the help of the unified superspace approach (Elcoro *et al.*, 2012 ▶).

**Figure 13:**
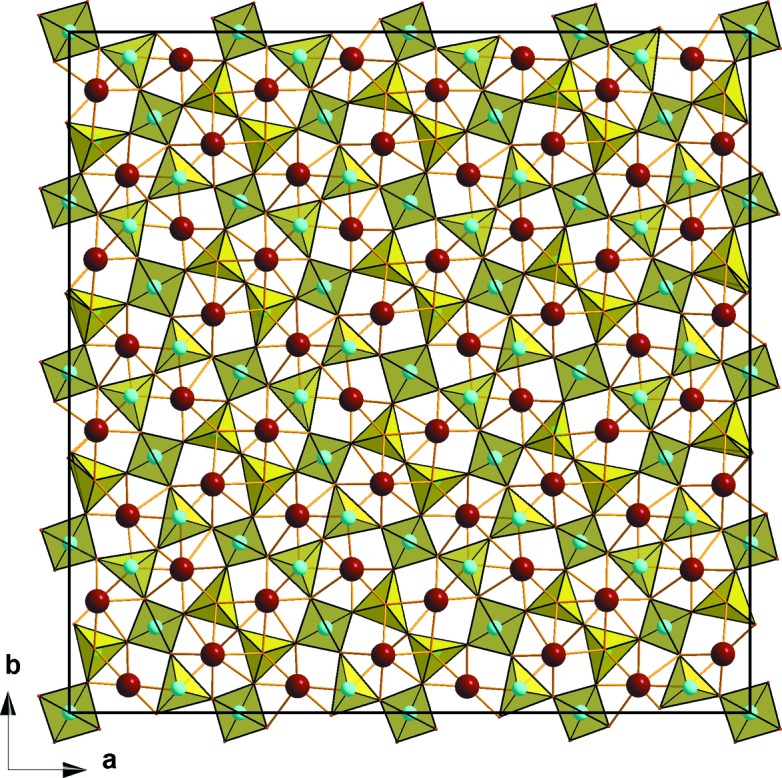
Crystal structure of the modulated [CaLa]_2_[Ga]_2_[Ga_2_O_7_]_2_ melilite. Ca and La cations are shown as brown spheres and Ga cations (light green) are situated in yellow oxygen tetrahedra. Note the variable number (6–8) of (Ca, La)–O bonds in the distorted pentagonal tunnels. The figure is drawn using the crystallographic data from Wei *et al.* (2012 ▶).

Incommensurate modulations of the anion sublattice are intrinsic in the oxide ion conductors with the melilite-type structure with the general composition [*A*_2_]_2_[*B*′]_2_[*B*″_2_O_7_]_2_, where *A* is large alkali-earth or rare-earth cations, *B*′ and *B*″ are small tetrahedrally coordinated cations (Si^4+^, Ga^3+^, Ge^4+^ *etc*). The *B*′O_4_ tetrahedra share common corners with the *B*″_2_O_7_ tetrahedral dimers and form layers with distorted pentagonal windows. The stacking of the layers creates pentagonal tunnels occupied with the *A* cations. The ionic conductivity in melilites occurs due to interstitial oxygen atoms which arise upon heterovalent *A* ^2+^ → *A* ^3+^ doping and reside in the pentagonal windows (Kuang *et al.*, 2008 ▶). The incommensurability of the melilite structure, however, is not due to ordering of these interstitial O atoms, but merely due to distortion of the tetrahedral network caused by a mismatch of the size of the pentagonal tunnels and the ionic radii of the *A* cations (Ohmasa *et al.*, 2002 ▶, and references therein). This displacive modulation is confined mainly to the deformations of the *B*′O_4_ tetrahedra and rotations of the *B*″O_4_ tetrahedra causing bending of the *B*″_2_O_7_ dimers (Bindi *et al.*, 2001 ▶; Wei *et al.*, 2011 ▶, 2012 ▶). As a result, the pentagonal rings become deformed giving rise to six-, seven- and eightfold coordinated positions for the *A* cations, thus accommodating the mismatch (Fig. 13 ▶). The incommensurability is not directly related to the ionic conductivity of melilites, although easily deformable tetrahedral networks might be a necessary prerequisite for enhanced mobility of the interstitial O atoms (Kuang *et al.*, 2008 ▶; Wei *et al.*, 2011 ▶, 2012 ▶).

**Figure 14:**
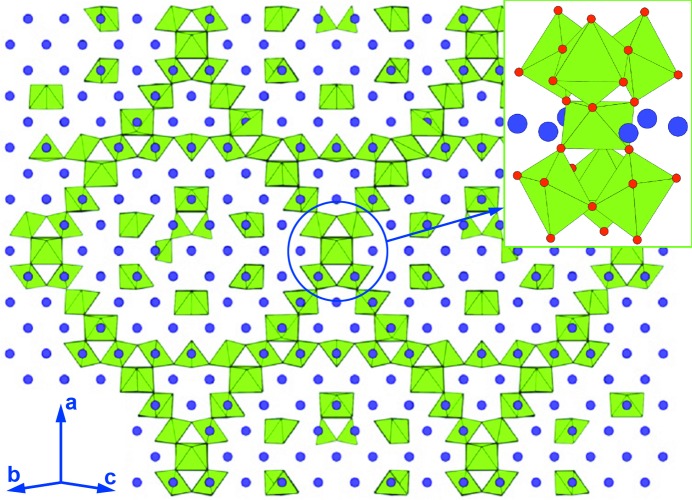
The 110 projection of the modulated (Bi_2_O_3_)_0.795_(Nb_2_O_5_)_0.205_ crystal structure. The Bi atoms are shown as purple spheres, the oxygen polyhedra around the Nb atoms are green. The insert shows the pyrochlore-like cluster of the NbO_6_ octahedra. Red spheres denote the oxygen atoms. The figure is adapted from Ling *et al.* (2013 ▶).

A spectacular incommensurate ordering of dopant cations, oxygen atoms and vacancies has been observed in a complex (3+3)-dimensionally modulated cubic fluorite-type structure of the Nb-stabilized δ-Bi_2_O_3_ phase, so far one of the best intermediate-temperature oxide-ionic conductors (Ling *et al.*, 2013 ▶). The chains of the corner-sharing NbO_6_ octahedra run along the 〈110〉 cubic directions forming the pyrochlore-type clusters of the NbO_6_ octahedra at the intersection points (Fig. 14 ▶). The octahedral chains delimit interpenetrating δ-Bi_2_O_3_-like channels with a high concentration of disordered oxygen vacancies around the Bi atoms, responsible for the high oxygen ion conductivity. The irregular and asymmetric coordination environment of the Bi^3+^ cations is attributed to the stereochemical activity of their lone electron pairs.

### 4.2. Modulations related to lone electron pair cations and anion deficiency   

The stereochemical activity of the lone electron pair is considered as a result of the interaction of the 6*s* states of cations such as Pb^2+^ and Bi^3+^ with the oxygen 2*p* orbitals filling both bonding and antibonding states, and the subsequent lowering the energy of the system by stabilizing occupied antibonding state through mixing with the cation 6*p* orbitals (Walsh *et al.*, 2011 ▶). This generates asymmetric electron density around the lone electron pair cation, which has a characteristic localized spatial distribution. Such orbital stabilization induces considerable displacement of the lone electron pair cation towards a limited number of oxygen atoms (typically two to four), thus shortening the corresponding interatomic distances and creating an asymmetric coordination environment.

**Figure 15:**
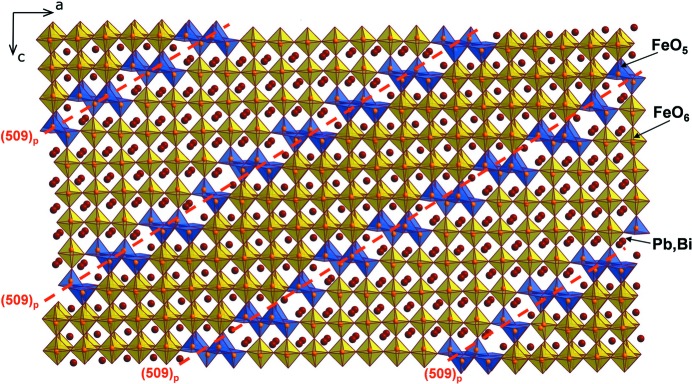
Crystal structure of the Pb_0.65_Bi_0.31_Fe_1.04_O_2.67_ perovskite modulated by ∼(509)_p_ crystallographic shear planes. FeO_5_ tetragonal pyramids and FeO_6_ octahedra are shown in blue and yellow, respectively.

Although intuitively the asymmetric coordination of the lone electron pair cations can favour the formation of oxygen vacancies, they can also act in the opposite way by eliminating point oxygen vacancies owing to the formation of the structures, which are incommensurately modulated by interfaces. These interfaces are similar to the crystallographic shear (CS) planes in the binary oxides derived from the ReO_3_ or TiO_2_ structures (Magneli, 1948 ▶; Kihlborg, 1963 ▶; Wadsley, 1955 ▶, 1961*a* ▶,*b* ▶; Andersson & Jahnberg, 1963 ▶). By doping the multiferroic BiFeO_3_ perovskite with the Pb^2+^ cations, oxygen deficiency is concomitantly introduced in order to maintain the charge balance. First, this deficiency is accommodated as disordered point vacancies (Chaigneau *et al.*, 2009 ▶). Then, at the Bi/Pb ≃ 4 ratio, an incommensurate short-range order sets in the pseudocubic Bi_0.81_Pb_0.19_FeO_2.905_ perovskite with uniformly spaced anion-deficient perovskite {001} (FeO_1.25_) planes approximately every six perovskite unit cells and transform every sixth layer of the FeO_6_ octahedra into a layer with a 1:1 mixture of corner-sharing FeO_4_ tetrahedra and FeO_5_ tetragonal pyramids (Dachraoui *et al.*, 2012 ▶). At the lower Bi/Pb ratio, the structure splits into quasi two-dimensional blocks by equidistantly spaced parallel translation interfaces, which can be considered as CS planes in the perovskite matrix (Bougerol *et al.*, 2002 ▶; Abakumov *et al.*, 2006 ▶). In such a long-range ordered incommensurately modulated (Pb,Bi)_1−*x*_Fe_1+*x*_O_3−*y*_ series, the perovskite blocks are separated by the CS planes confined to nearly the (509)_p_ perovskite plane (the subscript p refers to perovskite subcell) (Abakumov *et al.*, 2011 ▶). Along the CS planes, the perovskite blocks are shifted with respect to each other over the 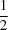
[110]_p_ vector that transforms the corner-sharing connectivity of the FeO_6_ octahedra in the perovskite framework into the edge-sharing connectivity of the FeO_5_ distorted tetragonal pyramids at the CS plane, thus reducing the oxygen content (Fig. 15 ▶).

The formation of CS planes in the perovskite structure requires a substantial rearrangement of the coordination environment of the lone electron pair cations. The corner-sharing FeO_6_ octahedra and edge-sharing FeO_5_ pyramids form six-sided tunnels with a very asymmetric coordination environment suitable for the lone electron pair cations. The oxygen content in the series is varied through changing the Bi/Pb ratio which affects only the thickness of the perovskite blocks, whereas the orientation of the CS planes almost does not change. This behaviour differs from that of the CS planes in the ReO_3_-based structures, where the orientation of the planes depends on the oxygen deficiency. In perovskites, the orientation of the CS planes is altered when the lone electron pair cations Pb^2+^ and Bi^3+^ are partially substituted by alkali-earth cations (Ba^2+^ or Sr^2+^) occupying the *A* positions in the perovskite blocks between the CS planes. Such a replacement can be used to stabilize the (101)_p_ CS planes resulting in the *A*_*n*_ *B*_*n*_O_3*n*−2_ perovskite-based homologous series, where the chemical composition changes by varying the thickness of the perovskite blocks between the CS planes (Abakumov *et al.*, 2010 ▶).

The electronic configuration *d* ^5^ of the Fe^3+^ cations does not imply polar off-centre displacements, contrary to the *d* ^0^ transition metal cations which are prone to such displacements owing to the second-order Jahn–Teller effect. Nevertheless, in the (Pb,Bi)_1−*x*_Fe_1+*x*_O_3−*y*_ perovskites the strain field created by periodic arrangement of the CS planes promotes strong off-centring for the Fe^3+^ cations, also supported by concomitant displacements of the lone electron pair cations. This creates local polarity, but the polarization directions are opposite on both sides of the CS planes resulting in an antipolar structure. Thus, the Pb-doping of the ferroelectric BiFeO_3_ perovskite first leads to the paraelectric pseudocubic phase with a short-range order of point oxygen vacancies and then to the antiferroelectric-type incommensurately modulated structure.

### 4.3. Modulations due to off-centre displacements and octahedral tilting in perovskites   

**Figure 16:**
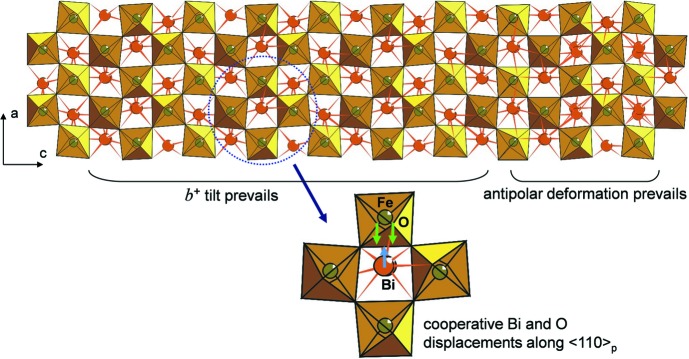
Incommensurately modulated crystal structure of Bi_0.75_La_0.25_FeO_3_ perovskite.

Diluting the lone electron pair Bi^3+^ cations in BiFeO_3_ by stereochemically inactive rare-earth cations (La^3+^) also results in an incommensurate ordering. The La for Bi replacement transforms ferroelectric BiFeO_3_ into paraelectric GdFeO_3_-type Bi_0.5_La_0.5_FeO_3_ through the incommensurately modulated antipolar Bi_0.75_La_0.25_FeO_3_ phase (Rusakov *et al.*, 2011 ▶). The primary modulation in this structure occurs due to a displacement of the Bi cations and a part of the O atoms towards each other along the 〈110〉_p_ perovskite direction. These displacements are ordered in an antipolar manner. The secondary modulation arises from a frustration of the octahedral tilt components. In *AB*O_3_ perovskites, the corner-sharing *B*O_6_ octahedra forming a framework are subjected to cooperative tilts and rotations (tilting distortion) in order to relieve a mismatch between the *A*—O and *B*—O interatomic distances. The cooperative character of this distortion limits the number of possible tilt patterns, which are denoted using the Glazer notation marking the magnitude and the direction (in-phase or out-of-phase) of octahedral rotations along the [100], [010] and [001] axes of the perovskite subcell [see Howard & Stokes (1998 ▶) for details]. The transitions from the *a* ^−^ *a* ^−^ *a* ^−^ tilt system in BiFeO_3_ into the *a* ^−^ *b* ^+^ *a* ^−^ tilt system in Bi_0.5_La_0.5_FeO_3_ occur *via* the modulated Bi_0.75_La_0.25_FeO_3_ phase where the *b* ^+^ component develops in a frustrated manner being coupled through the lattice strain to the antipolar atomic displacements. As a result of such competing interactions, the structure becomes split into the the regions where either the *b* ^+^ octahedral tilting component or the antipolar displacements prevail (Fig. 16 ▶).

Competing with antiparallel displacements of the lone electron pair cations, octahedral tilts and octahedral deformations also cause antiferroelectric-type incommensurate modulation in the Pb_2_CoWO_6_ perovskite (Baldinozzi *et al.*, 2000 ▶; Bonin *et al.*, 1995 ▶). Even without the lone electron pair cations, a similar competition between the cooperative octahedral tilting distortion and off-centre antipolar displacements of the Ti^4+^ cations are at the origin of complex (3+2)-dimensional incommensurately modulated structures in the Li_3*x*_­Nd_2/3−*x*_TiO_3_ perovskites (Abakumov *et al.*, 2013 ▶; Erni *et al.*, 2014 ▶; Davies & Guiton, 2014 ▶; Guiton & Davies, 2007 ▶).

### 4.4. Modulations related to charge density wave, charge and orbital ordering   

Periodic lattice deformation can arise in quasi one-dimensional and two-dimensional systems owing to Peierls distortion introducing a gap in a partially filled conduction band. The lattice distortion lowers the energy of the filled state of the conduction band and gives rise to a charge density wave (CDW) with the same periodicity as the lattice deformation. Particular nesting conditions of the Fermi surface define the periodicity of the modulation which can be incommensurate with the underlying structure occurring above the transition to the CDW state. The modulation vector is defined by the reciprocal lattice vector connecting the parallel parts of the Fermi surface. Among inorganic materials, the low-dimensional systems demonstrating transitions to low-temperature incommensurate CDW state were found in chalcogenides, oxides, silicides and some others (van Smaalen, 2005 ▶, and references therein). Incommensurate CDWs appear to compete with superconductivity in layered cuprates (Ghiringhelli *et al.*, 2012 ▶; da Silva Neto *et al.*, 2014 ▶; Chang *et al.*, 2012 ▶) and there are indications that the CDW states might also appear in the half-doped manganite La_0.5_Ca_0.5_MnO_3_ with the framework perovskite structure (Cox *et al.*, 2008 ▶). An example of the oxide incommensurate CDW system is given by the monophosphate tungsten bronzes (PO_2_)_4_(WO_3_)_2*m*_. These structures are built of the regular stacking of WO_3_ slabs with a ReO_3_-type structure separated by layers of phosphate (PO_4_) groups. The thickness of the WO_3_ slab increases with *m*, reaching the tungsten trioxide structure WO_3_ at the limit of *m* = ∞. The crystal structures of the members of this series with different *m* above the transition to the CDW state have been rationalized in superspace as *commensurately* modulated using a unified superspace model (Pérez *et al.*, 2013 ▶). Each phosphate group donates one electron to the WO_3_ slabs thus providing 2/*m* electrons per W atom and resulting in a metallic behaviour. Low-dimensional bands of the WO_3_ slabs cause CDW instabilities resulting in a transition into an incommensurately modulated state. The incommensurate CDW structures of the *m* = 4 and *m* = 10 compounds have been refined; in both cases, the modulation is due to two-dimensional CDW confined to the displacements of the W atoms (Lüdecke *et al.*, 2000 ▶, 2001 ▶; Roussel *et al.*, 2000 ▶).

Similar to a CDW, a cooperative structure distortion with a pairing of interatomic distances along chains of spin *S* = 1/2 entities originates from an enhancement of exchange interactions between neighbouring magnetic atoms (spin-Peierls dimerization). It gives rise to incommensurate modulations caused by a frustration between the spin-Peierls dimerization and elastic interchain coupling, as observed in TiOHal (Hal = Cl, Br) (Schönleber *et al.*, 2006 ▶; van Smaalen *et al.*, 2005 ▶) and TiPO_4_ (Bykov *et al.*, 2013 ▶).

**Figure 17:**
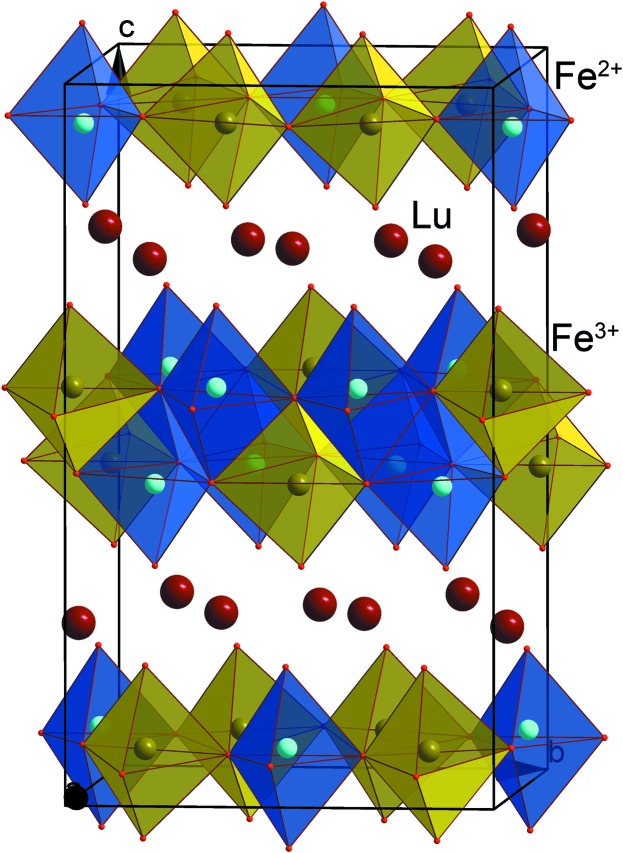
Crystal structure of LuFe_2_O_4_ in the charge ordered state. Oxygen polyhedra around the Fe^2+^ and the Fe^3+^ cations are shown in blue and yellow, respectively.

Charge ordering, implying electron localization in the form of a pattern of alternating cationic species with different formal valences, plays an important role in the physics of the strongly correlated electron systems. It has been proposed as a possible mechanism for inducing ferroelectricity in magnetic oxides, exemplified, among others, by LuFe_2_O_4_ (Ikeda *et al.*, 2005 ▶; van den Brink & Khomskii, 2008 ▶). This material exhibits a layered structure, consisting of alternating (LuO_2_) blocks and (Fe_2_O_2_) bilayers. The average iron valence in this compound is +2.5, and the Fe^2+^/Fe^3+^ charge ordering at ∼330 K has been supposed to induce electric polarization. Incommensurate ordering of the Fe^2+^ and the Fe^3+^ cations has been detected in this compound with electron diffraction, neutron and X-ray diffraction, and plausible models of this ordering were proposed to explain ferroelectricity (Ikeda *et al.*, 1998 ▶; Yamada *et al.*, 2000 ▶; Zhang *et al.*, 2007 ▶). However, recent refinement of the charge-ordered LuFe_2_O_4_ crystal structure in a *commensurate* approximation revealed that the arrangement of the different Fe valence states creates a nonpolar centrosymmetric structure, thus questioning the ‘ferroelectricity from charge order’ mechanism (de Groot *et al.*, 2012 ▶). Instead of the equally charged polar (Fe_2_O_2_) bilayers, the charge ordering induces a charge transfer between the adjacent bilayers retaining their nonpolar character (Fig. 17 ▶).

An example of a locally polar structure, but antiferroelectric on the long scale owing to incommensurate modulation, is Bi_2_Mn_4/3_Ni_2/3_O_6_, where lone pair-driven Bi displacement, chemical order of Mn and Ni and both charge and orbital order at the transition metal sites compete with each other (Claridge *et al.*, 2009 ▶). The average oxidation state of Mn in this compound is +3.5, which is known to demonstrate a very strong tendency to charge separation. The modulation arises owing to coupling of the local displacements of the Bi^3+^ cations and the distortions associated with electronic ordering at the octahedral *B* site, related to different structural environments of the Mn^4+^, Mn^3+^ and Ni^2+^ cations.

**Figure 18:**
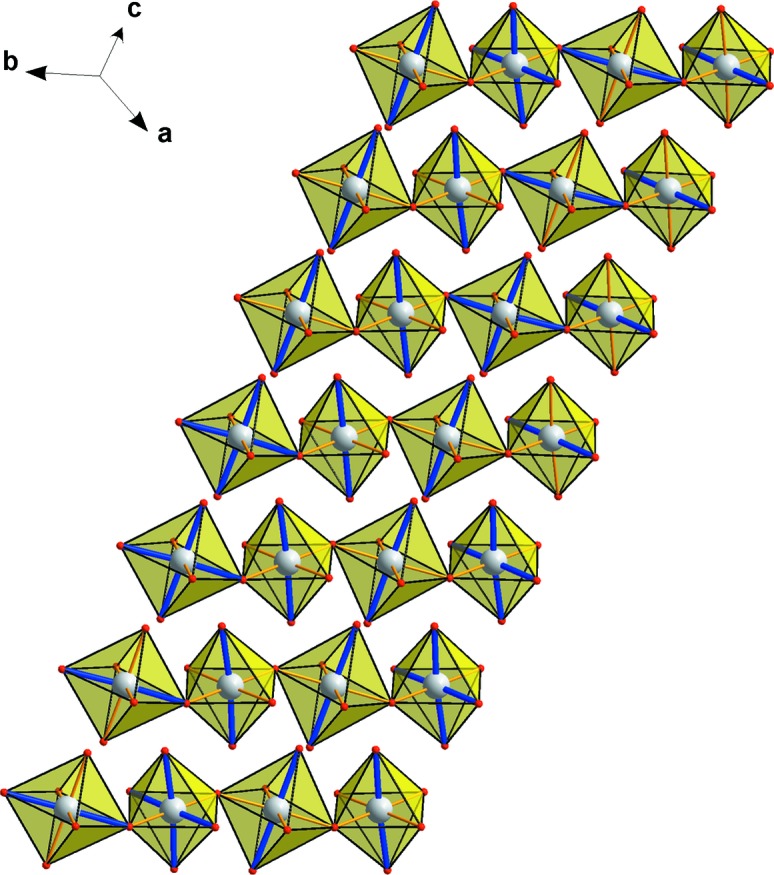
Fragment of the modulated CaMn_7_O_12_ crystal structure showing the pattern of the short (1.89–1.95 Å, thin yellow lines) and the long (2.05–2.13 Å, thick blue lines) Mn_*B*_^3+^—O bonds. Mn and O atoms are shown as grey and red spheres, respectively. The figure is drawn using the crystallographic data from Sławiński *et al.* (2009 ▶).

Along with the charge ordering, orbital ordering is a manifestation of electronic correlations in transition metal oxides. Multiferroic CaMn_7_O_12_ has a rhombohedrally distorted perovskite structure, where three-quarters of the *A* positions are occupied by the Mn^3+^ cations in a nearly square-planar oxygen coordination (Mn_*A*_) and the *B*O_6_ octahedra are taken together by the Mn^3+^ and Mn^4+^ cations (Mn_*B*_), which demonstrate a tendency towards charge ordering. Below *T* = 250 K, the incommensurate modulation wave propagating along the *c* axis causes strong variation of the Mn_*B*_—O interatomic distances, whereas the spread of the Mn_*A*_—O distances remains only weakly modulated (Sławiński *et al.*, 2009 ▶). The modulation can be interpreted as an incommensurate rotation of a pair of the Mn_B_^3+^—O long bonds of the Jahn–Teller-distorted Mn^3+^O_6_ octahedra with a concomitant switching of the population of the 3*x* ^2^ − *r* ^2^ and the 3*y* ^2^ − *r* ^2^ *d*-orbitals (four short and two long Mn—O bonds) through the intermediate *x* ^2^ − *y* ^2^ state (four long and two short Mn—O bonds), denoted as orbital rotation (Perks *et al.*, 2012 ▶) (Fig. 18 ▶). Below *T*_N_ = 90 K, this spiral orbital ordering is coupled with the spiral arrangement of the ordered magnetic moments (Sławiński *et al.*, 2012 ▶) resulting in large magnetically induced electrical polarization (Perks *et al.*, 2012 ▶).

### 4.5. Modulations due to cation-vacancy ordering   

The last example of an intimate interplay between incommensurability and functional properties is provided by the complex molybdates and tungstates with the scheelite-type (*A*′,*A*′′)_*n*_[(Mo/W)O_4_]_*m*_ structures, where *A*′,*A*′′ = alkali, alkaline-earth or rare-earth elements. The CaWO_4_ scheelite structure is built up by […–*A*O_8_–WO_4_–…] columns of the *A*O_8_ polyhedra and WO_4_ tetrahedra, which share common vertices and form a three-dimensional framework. Very often the occupation of the *A* sites in the scheelite-type structure by cations with different sizes and charges (such as alkali metal cations and rare-earth cations) and/or cation vacancies leads to modulated structures with a pronounced occupational modulation. The Eu^3+^-doped scheelites demonstrate strong red luminescence dominated by the ^5^D_0_–^7^F_2_ transition at 612 nm. Remarkably, the quantum yield and other luminescence parameters correlate not only with the overall Eu content, but also with the parameters of the occupational modulation defining ‘clustering’ of the Eu cations. In Na_*x*_Eu_(2−*x*)/3_MoO_4_ series the quantum yield raises concomitantly with the increasing number of the diatomic clusters with the Eu–Eu separation of ∼3.95 Å (Arakcheeva *et al.*, 2012 ▶).

## 5. Conclusion   

In this brief review, we have tried to outline the importance of the modulated structures for the understanding of interatomic and intermolecular interactions in crystalline solids, the related chemistry and, ultimately, the functional properties.

Investigation of molecular modulated complexes helps to improve their structural description and their role in the interplay between molecular configurations and crystal packing. Indeed, most of the molecular modulated complexes present steric effects and weak intermolecular interactions pointing towards different configurations in the solid state. This competition often results in a change of conformation of one or more flexible parts of the molecules. There are also examples relating modulation to the acidity change and the accommodation of guest structures in host hydrogen-bonded networks. Nevertheless, the total number of reported modulated organic structures is still small and the role of the modulation in the crystal packing in a broad framework is not yet clear.

Knowing the driving forces behind incommensurability should help chemists to find pathways in order to avoid or diminish the possible detrimental effects of such ordering. Alternatively, this knowledge might help in achieving and improving the desired functionality, or even generate combined functionalities, such as multiferroicity arising from incommensurate cycloidal magnetic ordering, which in turn gives rise to ferroelectricity (Cheong & Mostovoy, 2007 ▶). It would be tempting to advocate that the paradigm has to be shifted from ‘superspace crystallography’ towards ‘superspace chemistry’ or even to ‘superspace material science’, which will be based on targeted implication of modulations of different types and nature for the design of compounds with the required chemical composition, crystal structures and functional properties. A crucial step would be linking together the superspace crystallography and computational methods, which will be of tremendous help in unraveling the role of relatively weak, frustrated and/or competing interactions in the formation and stability of the modulated structures.

Finally, we would like to comment on two major challenges that seem to be an obstacle to more intense investigation of modulated crystals. The first one is purely experimental and related to the need for a wide dissemination of the superspace crystallography among young researchers, as well as among more experienced crystallographers. In our nonscientific opinion (as there will be no data on this subject!), in many cases the incommensurability of the crystals has been overlooked as twins or even discarded as poor crystals. The second challenge is the obvious need of a deeper understanding of the basic formalism of the superspace approach and its practical use. Citing a colleague who tried to address this problem before ‘…while crystallography might be a black box for some, superspace crystallography is a black art for others’ (Christensen, 2010 ▶). Here the options are to disseminate the knowledge through schools, workshops, seminars and online resources and further promoting the basic references on the subject (*e.g.* Janssen *et al.*, 2006 ▶, 2007 ▶ and van Smaalen, 2007 ▶), or to pursue the efforts like the one already undertaken by Wagner & Schönleber (2009 ▶) to explain to beginners, in the most accessible possible way, the superspace approach.

## Footnotes

1 The average structure is a three-dimensional periodic structure with atoms at the positions obtained by averaging all their positions in the modulated structure.

2 An introduction and explanation of superspace group symbols can be found in Janssen *et al.* (2006 ▶) and van Smaalen (2007 ▶).
